# Silicon Dioxide Nanoparticles and Biochar to Suppress Leaf Blight and Fruit Rot in Eggplant

**DOI:** 10.3390/jof12050300

**Published:** 2026-04-22

**Authors:** Masudulla Khan, Lukman Ahamad, Younes Rezaee Danesh, Ivana Castello, Gaetano Iacono, Alessandro Vitale

**Affiliations:** 1 Botany Section, Women’s College, Aligarh Muslim University, Aligarh 202002, India; masudkhann@gmail.com; 2 College of Agriculture, Guizhou University, Guiyang 550025, China; lukmanamu@gmail.com; 3 Department of Plant Protection, Faculty of Agriculture, Van Yuzuncu Yil University, Van 65090, Türkiye; 4 Department of Agriculture, Food and Environment, University of Catania, 95123 Catania, Italy; castelloivana75@gmail.com (I.C.); iacono.gaetano@gmail.com (G.I.)

**Keywords:** *Phomopsis vexans*, biochar, SiO_2_-NPs, *Solanum melongena*

## Abstract

Leaf blight and fruit rot caused by *Phomopsis vexans* are critical issues for eggplant crops. Our study evaluated the biochar amendment, alone and in combination with a foliar spray of silicon dioxide nanoparticles (SiO_2_-NPs), on plant performance and disease development. Fungal infection reduced plant growth, with a 22% decline in plant height and a marked decrease in chlorophyll and carotenoid levels. Adding biochar plant height mitigated these effects: the highest dose (30 g) increased plant height in infected plants by 17.1% and increased pigment concentrations and POX and PPO activities. At the same time, the blight index declined. When biochar was combined with SiO_2_-NPs, the improvements were more pronounced. In infected plants, the 30 g + SiO_2_-NPs treatment produced substantial improvement in plant height (+31.3%) and shoot biomass and restored chlorophyll and carotenoid contents by 63% and 28.1%, respectively. This treatment also produced the lowest blight index and the strongest enzymatic responses. Principal component analyses discriminated treated plants from infected ones. These findings indicate that biochar and SiO_2_-NPs can jointly enhance plant resilience to *P. vexans* infection, reducing its negative impact.

## 1. Introduction

Eggplant is prone to various pathogens, some of which cause leaf blight and fruit rot that highly reduce the quality and quantity of crops. These diseases are a significant challenge to food security [1]. One of them is *Phomopsis vexans*, which is one of the fungal pathogens involved in leaf blight and fruit rot in eggplant, causing significant agricultural losses [2]. *P. vexans* attacks provoke damping-off, stem blight, and fruit rot. These impacts result in crop wilting, necrosis, and decreased marketability [3,4]. Environmental conditions are favorable and contribute to the diffuse nature of this pathogen; as such, strong and sustainable management approaches are necessary to reduce its effects. Conventional methods of managing diseases also tend to be heavily based on artificial pesticides. Nevertheless, the growing awareness of environmental pollution created the demand for more environmentally friendly and sustainable options [5,6]. To overcome these issues, nanotechnology and natural-based solutions are becoming a good solution to crop protection [7].

In the nanotechnology industry, nanoparticles (NPs) have demonstrated potential in the agricultural industry through their distinctive physicochemical characteristics, that is, tunable size and high surface-to-volume ratio, which enhance the delivery of nutrients and activate plant defenses [8]. Agricultural nanotechnology is coming up with a new generation of tools to manage plant diseases. Among them, silica, polymer mixes, and chitosan are the most popular carriers of nanoparticles [9]. Biosynthesized silica nanoparticles have shown significant antifungal effects against pathogens, including *Alternaria solani*, the cause of early blight disease in eggplant, among NPs [10].

In addition to the NPs, there is biochar, a pyrolysis product of biomass in the form of charcoal. Given the positive effects that this product has on microbial activity and retention of nutrients, it is used as a soil health improver, a carbon sequestration tool, and a control of plant diseases agent [1,11,12,13,14,15]. The biocontrol capacity of biochar is also directly associated with its porous architecture, which serves as a habitat to useful microorganisms, and thus promotes a favorable environment of antagonistic relationships against soil-borne pathogens [16]. Biochar has the potential to lower the occurrence of *Fusarium* wilt in different crops through the effects of microbiome composition in the soil and the resistance of plants [17]. The use of biochar and biopolymers from organic waste can help to manage soil-borne plant diseases caused by pathogenic fungi and bacteria, including *Fusarium oxysporum*, *Sclerotium rolfsii, Sclerotinia sclerotiorum, Rhizoctonia solani*, and *Ralstonia solanacearum* [5,18,19,20]. Biochar application to the soil was also effective in overcoming the blight of pepper caused by the pathogenic oomycete, *Phytophthora capsici* L. [21]. The alteration of soil chemistry due to biochar has a strong connection with the suppression of diseases in several soil-borne bacteria [20,22]. The control effect can be maximized with the use of biochar immediately before planting due to the short-term effect of biochar on the biological properties of soil [23,24]. Biochar has been reported to induce systemic plant defenses in *Botrytis cinerea* (gray mold) and *Podosphaera aphanis* (powdery mildew) in strawberry and *B. cinerea* (gray mold) in tomato, respectively [25,26]. In combination with other agents, e.g., nanoparticles, disease-suppressive effects might be enhanced many times, providing a synergistic treatment of integrated pest management [14].

Such merging of nanotechnology and biochar use is one of the highest-level approaches towards sustainable agriculture, which tries to use the unique benefits of each constituent to improve disease suppression and elevate crop yield [27,28]. In the present research, we assumed that the combined use of SiO_2_-NPs foliar application and biochar amendment of the soil might be a viable approach to manage *P. vexans* in eggplant. The purpose of this study was to evaluate the appropriateness of this mixture as a *Phomopsis* sustainable method for eggplant growth.

## 2. Materials and Methods

### 2.1. Plant Material

The seeds of eggplant (*Solanum melongena* L.) cultivar VNR-218 were purchased from the local seed market of Aligarh, U.P., India. The seeds of the test plant were surface sterilized with 1.5% NaOCl for five minutes, followed by rinsing thoroughly in distilled water (DW) to remove the chemicals present on the seed surface, and then dried on blotter paper and finally used for the experiment.

### 2.2. Preparation and Sterilization of Soil and Biochar Mixture

For the cultivation of eggplant, silt–loam and clay–loam soils were preferred, and a pH range of 5.5 to 6.0 of soil is suitable for growth (with a ratio of 2:1:1 *v*/*v*, loam soil: sand: farmyard manure). The experimental soil had a sandy loam texture. It was collected from the top 20 cm layer of the field of Aligarh Muslim University, India. The soil mixture was placed in jute bags, and water was poured into each bag to wet the soil. Jute bags were put in an autoclave for sterilization at 137.9 kPa for 20 min. Earthen pots of 30 cm diameter were filled with 3.5 kg of sterilized soil after the said soil had cooled down at room temperature. Plants were sown in the earthen pots filled with autoclaved soil and grown under greenhouse conditions (Photoperiod—16/8 h; Temperature—28/20 °C; and Relative Humidity (RH)—65%). Inoculation of the pathogen and sterilization of the soil were done as explained earlier in our previous study [29].

**Figure 1:**
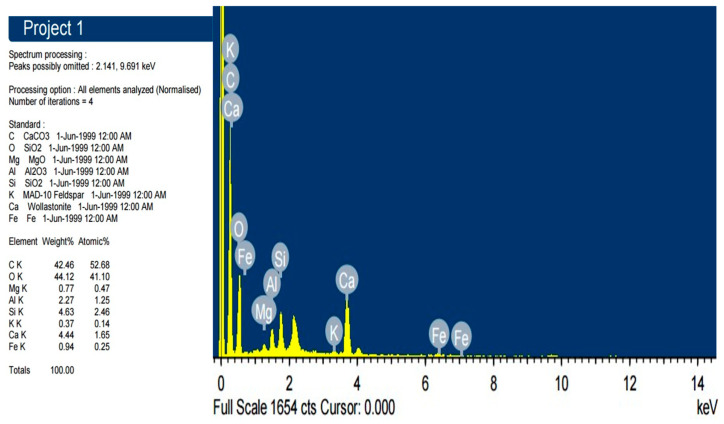
EDS analysis showing the elemental composition of Biochar.

Biochar was kindly provided by Casa De Amor (www.CasaGardenShop.com), India. The biochar was passed through a 40-mesh screen. The biochar was ground to a powder of <1 mm particles and stored in sealed containers until use. Biochar composition was analyzed by EDS spectrum (Figure 1).

### 2.3. Preparation of Fungal Inoculum

Leaves exhibiting disease symptoms were cut into small pieces and placed on Petri dishes containing potato dextrose agar (PDA) and incubated at 25 °C for 15 days. To obtain sufficient inoculum, fungi were grown in Richard’s liquid medium [30], which contained 10 g potassium nitrate, 5 g potassium dihydrogen phosphate, 2.5 g magnesium sulfate, 0.02 g ferric chloride, 50 g sucrose, and 1000 mL distilled water. Richard’s liquid medium was prepared, filtered through muslin cloth, and autoclaved for 15 min at 103.4 kPa in 250 mL Erlenmeyer flasks containing 80 mL of medium. After sufficient fungal growth, the liquid medium was filtered through Whatman filter paper No. 1. The fungal mycelia mat on the filter paper was washed in distilled water, and excess water and nutrients were removed with blotting paper. The inoculum was prepared by mixing 10 g of fungal mycelium in 100 mL of distilled water, and the mixture was blended (10,000 rpm) for 30 s in a Waring blender. Twenty mL of the suspension, containing 2 g of fungus, was used for the inoculation of seedlings [13,29,30].

### 2.4. Silicon Dioxide Nanoparticles (SiO_2_-NPs)

The SiO_2_ nanopowder (particle size 5–15 nm, spherical, porous, product number 637246-50G) was bought from Sigma Aldrich (Saint Louis, MO, USA). In our previous study [13], we screened the different concentrations of SiO_2_-NPs on eggplant alone and with *P. vexans.* We found that 0.2 mg L^−1^ was most effective. In the current study, we used the same concentration (0.2 mg L^−1^) of SiO_2_-NPs with biochar to observe the combined effect on the growth of eggplant alone and inoculated with the fungal pathogen (*P. vexans*). Spraying of SiO_2_-NPs was done on plants as explained in our previous study [13].

### 2.5. Experimental Design

Two weeks after germination, thinning was performed, and a healthy seedling was maintained in each pot. After thinning, 2-week-old, healthy, and well-established seedlings were used for the inoculation of pathogens and SiO_2_-NPs. A similar amount of water was poured into the control pots, in the same way around the roots. The experiment was conducted under greenhouse conditions. The experiment contains the following treatments:

• T1—Control
• T2—*P. vexans* fungus
• T3—10 g Biochar Alone
• T4—20 g Biochar Alone
• T5—30 g Biochar Alone
• T6—10 g Biochar + *P. vexans*
• T7—20 g Biochar + *P. vexans*
• T8—30 g Biochar + *P. vexans*
• T9—10 g Biochar + 0.2 mg L^−1^ SiO_2_ NPs
• T10—20 g Biochar + 0.2 mg L^−1^ SiO_2_ NPs
• T11—30 g Biochar + 0.2 mg L^−1^ SiO_2_ NPs
• T12—10 g Biochar + 0.2 mg L^−1^ SiO_2_ NPs + *P. vexans*
• T13—20 g Biochar + 0.2 mg L^−1^ SiO_2_ NPs + *P. vexans*
• T14—30 g Biochar + 0.2 mg L^−1^ SiO_2_ NPs + *P. vexans*

The experiment was laid out in a complete randomized design (CRD) with five replications of each treatment. The seedlings were inoculated with the conidial suspension of *P. vexans* (10^8^ conidia mL^−1^).

### 2.6. Structural and Agronomic Traits Assessments

Five plants of each biological replicate were used to calculate the growth parameters. Plant growth and physiological parameters were evaluated at the end of the experiments (120 days after transplanting). Plant height was measured from the stem base to the top. All eggplants, both inoculated and non-inoculated, were harvested for agronomically important traits analyses. All plants were carefully removed from the bases and placed on blotting paper to remove excessive moisture from the plants. After that, plant height was measured by using a centimeter scale. For plant dry weights, we cut the plant at the root initiation zone to separate roots and shoots by using a cutter. Subsequently, the roots and shoots of plants were kept in envelopes for drying at 60 °C in an Oven. The chlorophyll and carotenoid contents of the plants were recorded by Mackinney’s (1941) [31] method.

### 2.7. Disease Rating Assessment

The rating assessments were performed by analyzing the disease incidence (% infection of plants) and disease severity (damaged proportion of plant tissues) in all treatments. The plant disease severity was scored by using a 0 to 5 rating scale. Therefore, the leaf blight indices were determined by scoring disease severity on the visual appearances of disease symptoms. So, the disease ratings were calculated according to a 0-to-5 scale, where 0 = no disease (plants without leaf blight symptoms); 1 = leaf bight infection up to 12.5% on plant; 2 = blight infection up to 12.6–25%; 3 = blight infection up to 25.1–37.5%; 4 = leaf blight infection up to 37.6–50%, and 5 = greater than 50% leaf blight infection on plant.

### 2.8. Estimation of Peroxidase and Polyphenol Oxidase Enzyme Activities

The enzyme activity of peroxidase (POX, EC 1.11.1.7) was measured by the following method of Chance and Maehly [32] in fresh leaf samples. To a 3 mL solution of pyrogallol phosphate buffer, 0.1 mL of the enzyme extract, and 0.5 mL of 1% H_2_O_2_ were mixed in a cuvette, and a change in absorbance, at 20 s intervals for 3 min, was read at 420 nm on a spectrophotometer.

The activity of polyphenol oxidase (PPO, EC 1.14.18.1) in fresh leaves was determined using the procedure given by Mayer et al. [33]. The reaction mixture consisted of 200 μL of the enzyme extract and 1.5 mL of 0.1 M sodium phosphate buffer (pH 6.5). To start the reaction, 200 μL of 0.01 M catechol was added, and the change in absorbance was recorded at 30 s intervals up to 3 min at 495 nm on a spectrophotometer. The control set was prepared by all the above reagents except the enzyme extract. The activities of both enzymes, peroxidase (POX) and polyphenol oxidase (PPO), were expressed as U mg^−1^ FW.

### 2.9. Statistical Analysis

All the results are means of at least 5 replicates (mean ± SE). SigmaPlot 13.0 (Systa Software Inc., USA) was used to plot graphs. R (2.14.0) was used to analyze the gathered data. To determine if the applied treatments were statistically significant (*p* ≤ 0.05), analysis of variance (ANOVA) was used. To demonstrate significant changes between the treatments, Duncan’s multiple range test (DMRT) and least significant differences (LSD) were used. The standard error (±SE) is represented by error bars on the graphs. Principal component analysis was also used to analyze every observable parameter (PCA, OriginPro 2022).

## 3. Results

### 3.1. Effects of Biochar Alone on Plant Growth Attributes

**Figure 2:**
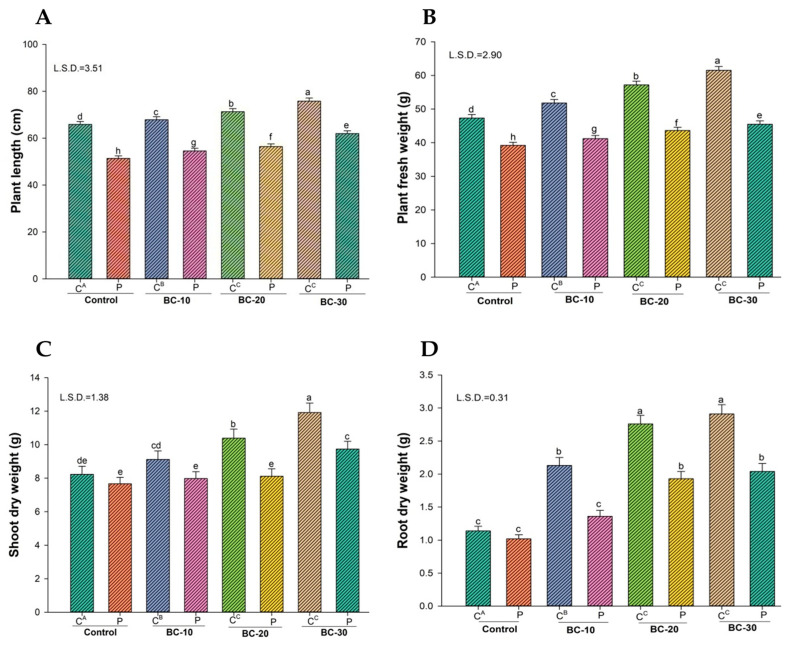
Effect of different concentrations of biochar on the plant growth of eggplant. Data analysis of significance was done by using Duncan’s multiple range test (DMRT) at *p* ≤ 0.05. Columns followed by the same letters indicate not significant difference. C, uninfected treatment; P, infected treatment; BC, biochar amendment at different rates (10, 20, and 30 g). (**A**) Plant length (cm); (**B**) Plant fresh weight (g); (**C**) Shoot dry weight (g); (**D**) Root dry weight (g).

The results of a greenhouse experiment revealed that all the tested concentrations of biochar were found effective against the leaf blight pathogen, *Phomopsis vexans*, in eggplant. Plants inoculated with *P. vexans* showed decreased plant growth parameters compared with the uninoculated control. However, inoculation of *P. vexans* to the plants caused 22% reduction in plant height over the control. We screened different biochar concentrations and reported concentration-based results. All biochar amendments in soil (10 g, 20 g, and 30 g) cause an increase in all growth parameters, but the highest increase in growth parameters was noticed in plants when treated with 30 g biochar, showing the concentration-based effect of biochar. The increase in plant height was observed at 13% in uninoculated plants after amending with 30 g biochar. However, an increase in plant height (17.1%) was observed in *P. vexans*-inoculated plants (Figure 2).

### 3.2. Effects of Biochar and Silicon Dioxide Nanoparticles (SiO_2_-NPs) on Plant Growth Attributes

**Figure 3:**
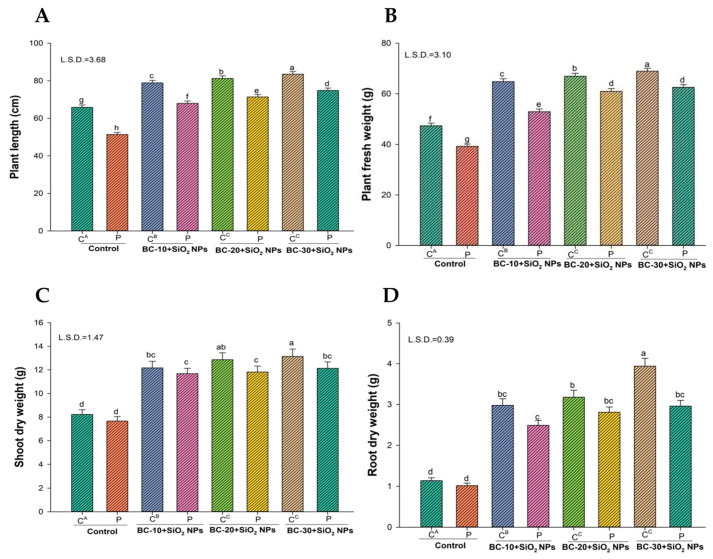
Effect of SiO_2_-NPs and biochar on the plant growth of eggplants. Data analysis of significance was done by using Duncan’s multiple range test (DMRT) at *p* ≤ 0.05. Columns followed by the same letters indicate not significant difference. (**A**) Plant length (cm); (**B**) Plant fresh weight (g); (**C**) Shoot dry weight (g); (**D**) Root dry weight (g).

With a target to develop a new effective formulation for fungal disease management. We applied SiO_2_-NPs and biochar together to know their cumulative impact. The combined use of biochar and SiO_2_-NPs showed better results in suppression of Phomopsis leaf blight in comparison to the control and biochar alone. Also, 10 g biochar and SiO_2_-NPs increase plant height by 16.4% in uninoculated plants and 24.3% in inoculated plants. The highest increase in uninoculated plant height (21.2%) was reported in 30 g biochar with silicon dioxide nanoparticles. *P. vexans*-inoculated plants showed an increase in plant height of 31.3%. The increase in shoot dry weight is 37.3% in uninoculated plants and 36.7% in inoculated plants (Figure 3).

### 3.3. Effects of Biochar Alone and with SiO_2_-NPs on Photosynthetic Pigments (Chlorophyll and Carotenoid Content) and Enzymes

Inoculation of plants with *P. vexans* caused a significant reduction in chlorophyll and carotenoid contents in comparison to the uninoculated control. However, the reduction in chlorophyll content (29.9%) and carotenoid content (15.4%) was noticed in pathogen-inoculated plants.

**Figure 4:**
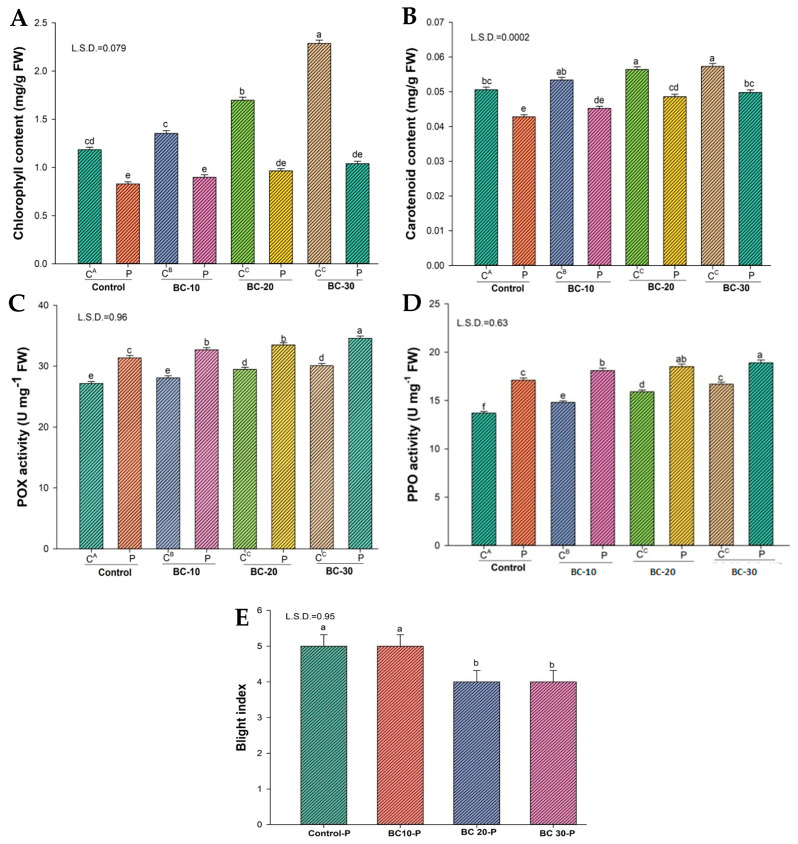
Effect of different concentrations of biochar on photosynthetic pigments of eggplant. Data analysis of significance was done by using Duncan’s multiple range test (DMRT) at *p* ≤ 0.05. Columns followed by the same letters indicate not significant difference. (**A**) Chlorophyll content (mg/g FW); (**B**) Carotenoid content (mg/g FW); (**C**) POX activity (U mg^−1^ FW); (**D**) PPO activity (U mg^−1^ FW); (**E**) Blight index.

**Figure 5:**
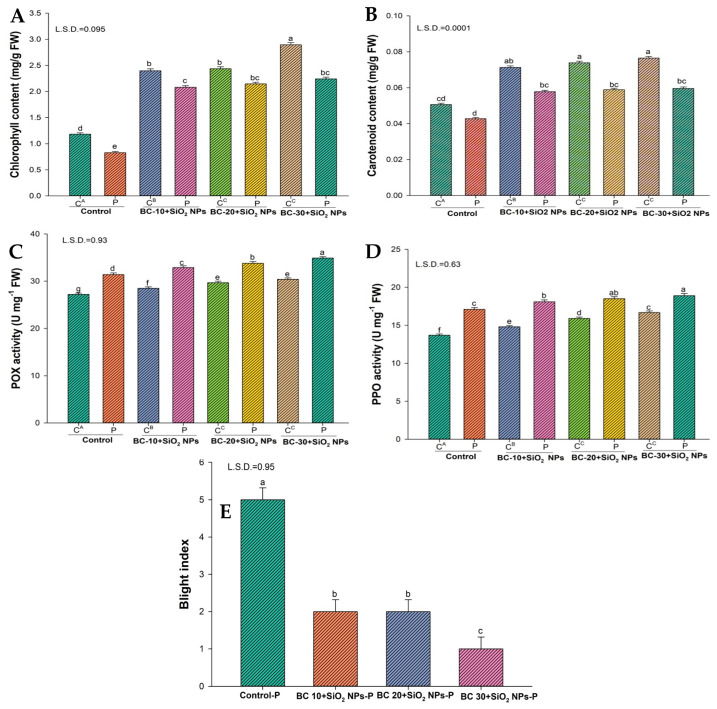
Effect of SiO_2_-NPs and biochar on photosynthetic pigments of eggplants. Data analysis of significance was done by using Duncan’s multiple range test (DMRT) at *p* ≤ 0.05. Columns followed by the same letters indicate not significant difference. (**A**) Chlorophyll content (mg/g FW); (**B**) Carotenoid content (mg/g FW); (**C**) POX activity (U mg^−1^ FW); (**D**) PPO activity (U mg^−1^ FW); (**E**) Blight index.

Biochar alone also improves chlorophyll and carotenoid content. In addition, the 30 g amendment causes the highest (among biochar alone) increase (48.2%) in chlorophyll content and an 11.6% increase in carotenoid content of uninoculated plants (Figure 4 and Figure 5). The highest increase in chlorophyll and carotenoid contents has been reported in plants treated with SiO_2_NPs and biochar (30 g). Chlorophyll (59.1%) and carotenoid (33.8%) contents increased in uninoculated plants after applying these treatments. Chlorophyll (63%) and carotenoid (28.1%) contents increased in inoculated plants after applying these treatments (Figure 4 and Figure 3). Peroxidase and polyphenol activity also increased after treatment with biochar alone and biochar with SiO_2_-NPs (Figure 4 and Figure 5). The highest increase in peroxidase (~17%) and polyphenol activity (~28%) occurs in biochar with SiO_2_-NPs in treated plants without a pathogen.

### 3.4. Principal Component Analysis (PCA)

**Figure 6:**
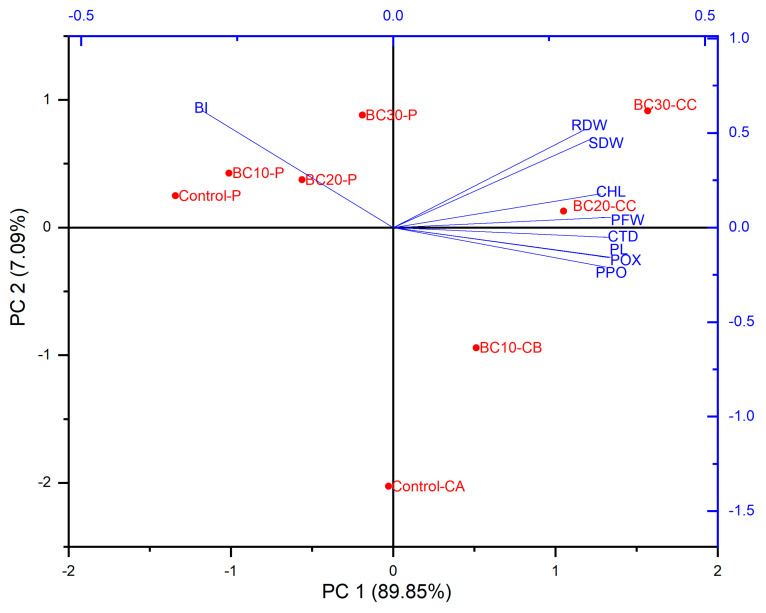
Principal component analysis (PCA) showing the effect of biochar on various studied attributes of eggplant infected with P = *P. vexans*; CA = Control, BI = blight index, BC10CB = 10 g Biochar alone, BC20CC = 20 g Biochar alone, BC30CC = 30 g Biochar alone, BC10-P = 10 g Biochar + *P. vexans*, BC20-P = 20 g Biochar + *P. vexans*, and BC30-P = 30 g Biochar + *P. vexans*.

**Figure 7:**
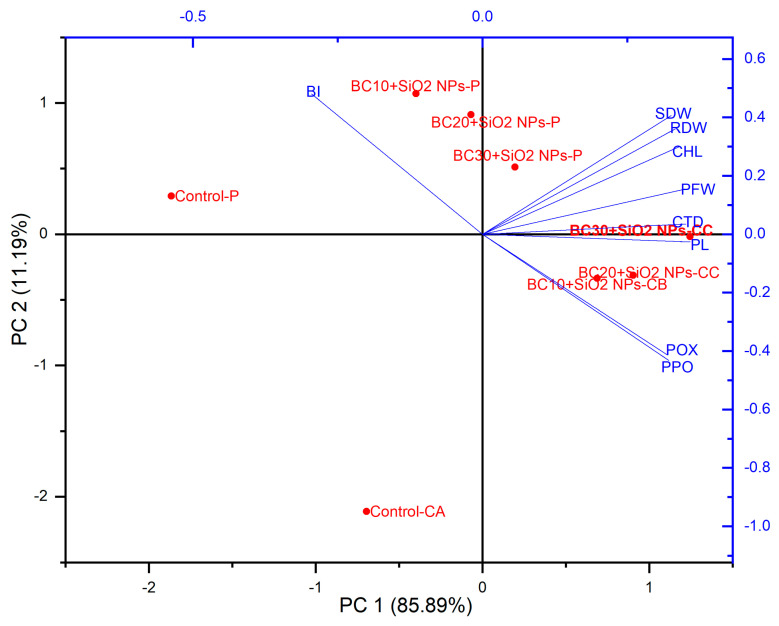
Principal component analysis (PCA) showing the effect of biochar and SiO_2_-NPs on various studied attributes of eggplant infected with P = *P. vexans*; CA = Control, BI = blight index, BC10 + SiO_2_-NPs-CB = 10 g Biochar + 0.2 mg L^−1^ SiO_2_-NPs, BC20 + SiO_2_-NPs-CC = 20 g Biochar + 0.2 mg L^−1^ SiO_2_-NPs, BC30 + SiO_2_-NPs-CC = 30 g Biochar + 0.2 mg L^−1^ SiO_2_-NPs, BC10 + SiO_2_ NPs-P = 10 g Biochar + 0.2 mg L^−1^ SiO_2_-NPs+ *P. vexans*, BC20 + SiO_2_-NPs-P = 20 g Biochar + 0.2 mg L^−1^ SiO_2_-NPs+ *P. vexans*, and BC30 + SiO_2_-NPs-P = 30 g Biochar + 0.2 mg L^−1^ SiO_2_-NPs + *P. vexans*.

Principal component analysis (PCA) was used to analyze the relationships between the plant parameters being studied. In the PCA biplot, it was observed that for biochar alone, PC1 accounted for a variation of 89.8% and PC2 for a variation of 7.09% (Figure 6), and in PCA for biochar and SiO_2_-NPs, PC1 accounted for a variation of 85.8% and PC2 for a variation of 11.1% (Figure 7). The analysis showed that treatments with biochar and SiO_2_-NPs had a significant impact on plant responses.

### 3.5. Effect of Biochar on Disease Suppression and Disease Indices

Regarding the disease indices caused by *P. vexans* in eggplants under greenhouse conditions, the blight indices were observed to be five when *P. vexans* was inoculated in plants. Application of the combined use of biochar with SiO_2_-NPs decreases the blight indices in plants (Figure 4 and Figure 5).

## 4. Discussion

The results of this study show that biochar, alone or combined with silicon dioxide nanoparticles (SiO_2_-NPs), substantially improve the growth, physiological performance, and defense responses of eggplant while reducing the severity of *P. vexans* under greenhouse conditions. These outcomes are consistent with the literature, demonstrating the capacity of biochar-based amendments to enhance plant vigor and contribute to disease suppression. The increases observed in plant height, biomass, chlorophyll, and carotenoid contents following biochar application reflect the well-established positive effects of biochar on soil structure, nutrient dynamics, and microbial activity. Previous works have shown that biochar improves plant performance in a range of species, including tomato, pepper, wheat, and strawberry, by enhancing nutrient retention, increasing cation exchange capacity, and stimulating beneficial microbial populations [34,35,36,37]. The present data align with these findings and indicate that similar benefits occur in eggplant grown in soils prone to infection by *P. vexans*.

In infected plants, biochar application consistently mitigated the negative effects of the pathogen. This pattern is comparable to observations in pepper affected by *Phytophthora capsici*, where biochar amendments reduced disease severity and improved plant physiological status [21]. Comparable reductions in pathogen impact have been reported in tomato subjected to *Fusarium* crown and root rot [38] and in strawberry exposed to *Botrytis cinerea* [25]. The partial recovery of chlorophyll and carotenoid levels observed in the present study suggests that biochar alleviated the physiological damage caused by *P. vexans*, a phenomenon that mirrors previous findings [29], which reported severe pigment degradation in diseased eggplants. By improving root-zone conditions and nutrient availability, biochar appears to help maintain photosynthetic functionality even when plants are under pathogen stress.

The increases in peroxidase (POX) and polyphenol oxidase (PPO) activities observed across biochar treatments suggest the activation of plant defense pathways traditionally associated with induced resistance. Similar enzyme responses have been reported in strawberry and tomato amended with biochar and exposed to fungal pathogens, where increased POX and PPO activities corresponded to enhanced systemic defense signaling [25,26]. These enzyme patterns are consistent with the broader literature on systemic acquired resistance (SAR) and induced systemic resistance (ISR), both of which rely on enzymatic responses associated with oxidative stress management and lignification [39,40].

The combined application of biochar and SiO_2_-NPs produced the greatest improvements across all parameters, particularly in infected plants. Silicon-based nanomaterials have been shown to enhance photosynthetic efficiency, stabilize chloroplast membranes, and improve water use and nutrient uptake, leading to higher chlorophyll levels and improved stress tolerance in wheat, maize, tomato, and lettuce [41,42,43]. The increases in pigment content recorded here, including chlorophyll increments exceeding 60% in infected plants, agree with earlier studies demonstrating that SiO_2_-NPs can counteract stress-induced pigment loss by enhancing leaf structure and antioxidant mechanisms [44,45]. With respect to disease mitigation, SiO_2_-NPs have shown suppressive effects against several pathogens, including *Alternaria solani* in eggplant [10], bacterial leaf spot in pepper [11], and *Fusarium* wilt in watermelon and tomato [12]. The significant decrease in blight index following the combined treatments in the present study is therefore consistent with a broader trend in which silicon-based nanoparticles reinforce plant defenses and limit pathogen progression.

Multivariate analysis reinforces these observations by showing clear separation between infected untreated plants and those receiving biochar or biochar–SiO_2_-NP treatments. Similar PCA-based distinctions have been reported in tomato treated with biochar under *Fusarium* stress, where PCA highlighted coordinated improvements in biomass, pigments, and defense enzymes [38]. The clustering of infected treated plants closer to uninfected groups in the present work confirms that the combined treatments shift the physiological profile of infected plants toward healthier conditions.

While research integrating biochar and nanoparticles remains limited, existing studies support the observed synergistic patterns. It was found that biochar–nanocomposite amendments improved drought tolerance and nutrient-use efficiency more effectively than individual amendments in barley [28]. Also, the biochar combined with nanoparticles increased microbial enzyme activity and improved soil biochemical functioning [27]. These findings point toward a consistent interaction in which biochar enhances nanoparticle stability, distribution, and bioavailability, thereby strengthening their beneficial effects on plant physiology and soil function.

By reducing disease severity and maintaining plant performance under pathogen pressure, biochar and SiO_2_-NPs have the potential to reduce the use of chemical fungicides, improving environmental outcomes and lowering production costs. Biochar also contributes to soil carbon sequestration due to its chemical stability and slow decomposition, thereby supporting climate mitigation strategies [34]. Improvements in soil microbial activity and nutrient cycling associated with biochar application are well documented [19,24], and the present findings support the idea that biochar could enhance long-term soil fertility and resilience in intensive horticultural systems. SiO_2_-NPs, when used at low concentrations, are considered among the least ecotoxic nanoparticles due to the natural abundance of silicon in soils and the limited mobility of silica nanoparticles [44,45]. Their integration with biochar, which can immobilize or modulate nanoparticle release, further contributes to environmental safety.

Moreover, iron oxide nanoparticles have the potential to effectively control *Fusarium oxysporum* in eggplants with a reduction in disease occurrence of over 70% at the most appropriate concentration [1]. In the same manner, it is demonstrated that silicon dioxide NPs can cause systemic resistance in vegetation, and it serves as the primary defense against numerous pathogens, among them the causes of bacterial leaf spot in peppers [11]. In addition, it has been noted that the application of chemogenic sulfur and silica nanoparticles could inhibit tomato and watermelon plants of *Fusarium* wilt and boost in planta nutrient accumulation, respectively, and strengthen the plant biomass [12]. The presence of this versatile utility highlights the promise of different types of NPs, such as silicon dioxide, as effective biopesticides against fungi in crops of economic value [13,14].

## 5. Conclusions

This study shows that biochar, alone or combined with silicon dioxide nanoparticles (SiO_2_-NPs), improves growth, physiological traits, and defense responses in eggplant and reduces the severity of leaf blight and fruit rot disease of eggplant. The combined treatment consistently produced the greatest improvements, suggesting complementary effects between soil enhancement by biochar and silicon-driven reinforcement of plant physiology. These results highlight the potential of integrated organic–nanotechnological strategies for sustainable disease management. Future research should validate these findings through multi-season field trials and by comparing different biochar feed stocks and nanoparticle dosages. Long-term assessments of soil biological and chemical changes will be essential to evaluate environmental safety. Mechanistic studies using molecular approaches could clarify how biochar and silicon interact with plant defense pathways. Integrating these amendments with other sustainable practices—such as microbial inoculants or reduced-input fertilization—may further increase crop resilience and broaden their applicability.

## Data Availability

The original contributions presented in this study are included in the article. Further inquiries can be directed to the corresponding authors.
